# Extending the study of playfulness in romantic life: Analyzing associations with attachment and jealousy in same-gender and opposite-gender couples

**DOI:** 10.1038/s41598-024-70979-2

**Published:** 2024-08-30

**Authors:** Kay Brauer, Rebekka Sendatzki, René T. Proyer

**Affiliations:** Martin Luther University Halle-Wittenberg, Emil-Abderhalden-Straße 26-27, 06108 Halle, Germany

**Keywords:** APIM, Playfulness, Attachment styles, Jealousy, OLIW, Romantic relationships, Human behaviour, Psychology

## Abstract

Adult playfulness describes individual differences in (re)framing situations so that they are experienced as entertaining, and/or interesting, and/or intellectually stimulating. There is increasing interest in its role for romantic life. Using the OLIW model of playfulness, we localized its facets Other-directed, Lighthearted, Intellectual, and Whimsical playfulness into systems of attachment styles and romantic jealousy. We analyzed data of 332 mixed-gender and 139 same-gender couples (*N*_total_ = 942). We found no robust mean differences between same-gender and mixed-gender couples (exception: same-gender couples are lower in emotional jealousy). Actor-Partner Interdependence Model analyses showed that Other-directed, Lighthearted, and Intellectual playfulness yielded negative relations to attachment insecurities in actors, but no partner effects. For jealousy, all types of playfulness related negatively to emotional jealousy, but Whimsical playfulness showed positive inclinations to cognitive and behavioral jealousy in actors. Partners reported greater cognitive jealousy when their partner was high in Lighthearted, whereas partners from Whimsical high scorers reported lower emotional jealousy. The findings are invariant between same-gender and mixed-gender couples. Our study extends the knowledge on how playfulness relates to experiences in close relationships in mixed-gender and same-gender couples. We discuss implications (e.g., Signal Theory of Playfulness) and future directions.

## Introduction

Adult playfulness is a personality trait that describes individual differences in how people (re)frame situations in a way that makes them interesting, and/or entertaining, and/or stimulating^1^. There is increasing interest in the study of playfulness in adults^2^, particularly regarding its role in romantic life. Recent studies have highlighted the role of playfulness in romantic relationships, for example, regarding partner similarities, relationship satisfaction, and love styles^3^. In the present study, we extended existing research in two ways: First, we examined the relationships between facets of playfulness and two distinct models describing individual differences in experiencing and behaving in romantic relationships: attachment styles and romantic jealousy; second, we examined these associations in mixed-gender and same-gender couples and tested the invariance of the findings between the samples. This study advances our understanding of the multifaceted nature of playfulness in romantic relationships. We explored how playfulness relates to internal models of closeness in relationships (*attachment*) and cognitive, emotional, and behavioral reactions to potential relationship threats (*jealousy*), thereby contributing to the broader network of factors related to playfulness.

### Adult playfulness

Although playfulness as a personality trait was acknowledged almost a century ago when, for example, Murray^4^ described the *need for play* in his theory of psychological needs (*personology*), systematic research on the structure and consequences of the trait has gained interest during the past decade^5^. Based on multi-methodological approaches, Proyer^1^ introduced the OLIW model, which describes four facets or types of playfulness: *O*ther-directed (e.g., using one’s playfulness to reduce social tension), *L*ighthearted (e.g., seeing life as a game rather than battlefield), *I*ntellectual (e.g., liking to play with ideas), and* W*himsical (e.g., liking unusual activities). The introduction of the OLIW model facilitated a fine-grained analysis of playfulness, including the malleability of its facets through short exercises in a randomized, placebo-controlled study design. It has also been used to examine differential expressions of playfulness across vocational groups and its reflection in language use. Furthermore, the OLIW model helped extend the understanding of the nomological net of playfulness by testing associations with indicators of creativity, well-being, and physical activity, and coping with stress among others^3,6–11^. Overall, the literature has shown that playfulness has an impact on the daily life of adults across a variety of life domains—including romantic relationships.

### Playfulness in romantic relationships

Playfulness has been studied in the domain of romantic relationships for more than four decades. Betcher^12^ highlighted the role of intimate play in bonding, trust, and intimacy after interviewing couples about their relationship experiences. Since then, research has repeatedly linked playfulness to various aspects of romantic life, including sexual preferences and sexuality^13–15^, the initiation of a relationship (e.g., mating processes), partner similarities, and relationship experiences (e.g., lower level of disagreements or conflict, better response to couple therapy, and using love idioms; see^3^ for an overview).

Chick’s^16^ *Signal Theory of Playfulness* proposes that playfulness might have a signal function in heterosexual couples seeking long-term relationships. High playfulness signals low aggressiveness in men, whereas playfulness in women signals fecundity. Overall, this notion has received support, as studies with samples from the United States, Switzerland, and Germany have found that playfulness is ranked among the most desired traits when participants are asked to describe their ideal partners for short- and long-term relationships^17–19^. Also, dyadic data from 77 and 218 couples supported the notion of assortative mating preferences, as partners were characterized by similarities both in the single facets and the profiles of the OLIW model facets^20,21^. These findings were replicated when studying 116 couples aged 50 years and older^22^.

Beyond describing how couples express and experience playfulness in their relationship, research has consistently shown a link between playfulness and relationship satisfaction. Initial studies found positive associations between playfulness, positive emotions, and satisfaction in individuals^12,23,24^, and findings were extended by analyzing data from couples using the Actor-Partner Interdependence Model (APIM^25^). The APIM allows one to disentangle associations between partners’ playfulness and their outcomes (e.g., relationship satisfaction) while controlling for the dependence between partners’ playfulness. It also provides information about actor effects (e.g., associations of playfulness and relationship satisfaction within-person) and partner effects (i.e., how playfulness relates to one’s *partner’s* satisfaction). Findings from young-, middle-, and old-age couples have revealed that particularly Other-directed and Intellectual playfulness have positive associations with relationship satisfaction that partially spillover to the partners’ satisfaction^21,22^. Conversely, partners of those with high expressions of Lighthearted playfulness have reported greater mistrust toward their partner.

Still, the recent research has not addressed how playfulness relates to basic models of experiences in romantic relationships. Specifically, the connections between playfulness and attachment styles (i.e., working models of relationships) and how playfulness relates to jealousy have remained unclear. This study aimed to narrow this gap in the literature.

### Romantic attachment

Attachment styles describe individual differences in working models of close relationships. These differences are described along two orthogonal dimensions: anxiety (worries about a relationship's stability and partner reliability) and avoidance (a tendency to feel emotionally distant and avoid intimacy^26^). While early research assumed that attachment styles are formed during early childhood through experiences with parents as primary caregivers and are stable over time and persons, subsequent research showed that attachment styles are malleable, and that adult attachment styles are specific in relation to romantic partners (see^26^ for an overview).

Attachment styles contribute to understanding how people experience their relationships, cognitively and emotionally. Both attachment dimensions relate differentially to outcomes such as love styles, self-esteem, and self-conceptions (e.g., avoidance relates to having a concept of the independent self and anxiety relates robustly to interdependent self-concept^27,28^). Moreover, attachment relates to several outcomes, including relationship satisfaction, and predicts objective data such as relationship status and having ever been in a romantic relationship^27,29–32^. Overall, those with insecure attachment styles (in the sense of high expressions of attachment anxiety and avoidance) are less likely to enter relationships, and those who do report low satisfaction frequently worry about the stability of their relationships and whether their love is reciprocated^29,32^.

Playfulness has been linked to facilitating trust, bonding, and intimacy in humans and animals alike, and one can argue that playfulness generally relates to greater attachment security (i.e., low avoidance and anxiety^29,33^). Similarly, the literature has argued that playful individuals adopt positive views on their relationships, as playfulness helps in training social skills, meeting the needs of others, and setting boundaries during childhood^3^. A previous study^24^ using a general measure of playfulness^34^ found no relationship between playfulness and insecurity (attachment anxiety). However, there was a positive correlation with attachment security, which reflects trust in one’s partner (i.e., the opposite of avoidance^35^). Proyer et al.^36^ examined the associations between the OLIW model facets and a screening questionnaire for maladaptive personality traits (the Personality Inventory for DSM-5^37^), which includes the domain of detachment. Detachment was negatively related to Other-directed and Lighthearted playfulness, suggesting that those types of playfulness are less characterized by insecure attachment. However, these findings must be interpreted cautiously, as detachment was conceptualized as a *pathological* trait and was only assessed with a screening instrument that does not cover the breadth of the attachment construct.

To our knowledge, no study had yet localized the OLIW model facets of playfulness in romantic attachment styles. We expected that Other-directed playfulness would be characterized by a secure attachment style in terms of negative associations with avoidance and anxiety because those high in Other-directed playfulness use their playfulness to solve interpersonal tension. It could be argued that lower worries and greater trust in relationships (i.e., secure attachment) would go along with inclinations to use playfulness to cheer others up and practice sensitivity in interpersonal relationships. We expected negative associations between Lighthearted playfulness and attachment anxiety. Those high in Lighthearted playfulness tend to worry less in their everyday lives, and we expected this to translate into fewer cognitive concerns about relationship stability. We examined the associations with Intellectual and Whimsical playfulness in an exploratory fashion.

### Romantic jealousy

Jealousy describes individual differences in how people experience and react to perceived and/or objective threats to their romantic relationship. Pfeiffer and Wong^38^ introduced a model of jealousy that distinguishes three components: cognitive (i.e., worrying and thinking about threats to the relationship), emotional (i.e., affective reactions to threats), and behavioral (i.e., seeking evidence of threats, such as going through clothes of the partner without their knowledge) expressions of jealousy in relationships. Robust evidence shows that jealousy impacts how people experience relationships; for example, it can heighten worry about a partner's fidelity. At elevated levels, this could lead to behaviors that damage trust, such as searching a partner's belongings or messages for signs of infidelity^38,39^. Mixed findings have been reported concerning its association with relationship satisfaction. Some researchers propose that jealousy, when expressed in response to perceived threats, can indicate a partner's care and investment in the relationship, potentially relating positively to satisfaction. However, most studies have found a negative correlation, suggesting jealousy is linked to lower satisfaction for both the jealous person (*actor*) and their partner^40^.

To our knowledge, no study had yet examined the associations between playfulness and jealousy. Taking prior findings on love styles and playfulness into account^20^, the love style of Mania (i.e., obsessive love and needing reassurance that the partner provides love^41^) is of particular interest, as it shares conceptual and empirical overlap with jealousy^39^. Proyer et al.’s^20^ study showed that playfulness is unrelated to this love style in actors and partners. Additionally, it was noted that playfulness fosters bonding, and it could be argued that this contributes to actors showing less reactions to perceived and actual threats to their relationships. Conversely, it might be argued that the reframing process that characterizes playfulness^1^ could be “overexpressed” and might elicit worries about the relationship, triggering imagination of the partner’s infidelity. Some preliminary findings from the clinical domain showed that patients with increased anxiety levels showed higher playfulness^42^. Similarly, it might be argued that those with high playfulness could be more inclined to reframe relationship-related situations in a way that provokes perceptions of a threat to relationships, and, thus, to jealousy in actors.

Prior dyadic studies using APIM analyses found that *partners* of those with higher expression of Lighthearted playfulness reported greater mistrust concerning their partner^21,22^. Also, it has been argued that the preference for improvisation and less planning in Lighthearted high scorers might be perceived by their partners as being less reliable and committed to the relationships, thus being somewhat likely to potentially cheat on their partner^3^. Furthermore, the APIM studies of playfulness and satisfaction indicated a positive relationship between Whimsical playfulness and partners’ mistrust, but these did not reach statistical significance (*p*s = 0.058 and 0.061), and our study aimed at clarifying the role of Whimsical in such inclinations by testing the associations with the three fine-grained facets of jealousy. Given the conceptual similarities between what is assessed as mistrust in prior studies, namely, worrying about the partners’ fidelity, and Pfeiffer and Wong’s^38^ jealousy model, we expected to find associations with the cognitive dimension of jealousy.

### The present study

Our study investigated the associations between four facets of playfulness, romantic attachment, and experiences of romantic jealousy in couples. We collected dyadic data and utilized the APIM to analyze the relationships between these factors within and across partners. To examine whether our findings were invariant across different relationship types, we collected data from two independent samples, including same-gender and mixed-gender couples. Previous research had shown that actor- and partner-effects of playfulness are invariant for men and women, suggesting that gender did not influence the relationship between playfulness and romantic outcomes. However, thus far, data was only available for mixed-gender couples. We collected the first data on playfulness in same-gender couples, allowing us to examine gender invariance within and between same- and mixed-gender partner constellations and to expand our understanding of playfulness in same gender-relationships. Since this is the first study of playfulness in same-gender couples, we assumed invariance between same-gender and mixed-gender couples as null hypothesis and tested deviations from H0 in exploratory fashion, assuming the conventional 5% type-I-error rate.

## Methods

### Participants

We collected data from two samples in German-speaking countries. The mixed-gender sample comprised 332 couples (*N* = 664 participants) with a mean age of 29.3 years (*SD* = 11.8). The couples were together for an average of 6.8 years (*SD* = 9.7). We also tested 139 same-gender couples (*N* = 278 women). Their mean age was 27.3 years (*SD* = 7.4), and they were on average together for 2.9 years (*SD* = 3.7). The educational status was high in both samples, with about 40% holding a high school diploma qualifying them to attend university, and 40.5% (mixed-gender) and 47.8% (same-gender) held a university degree. About half of each sample were university students (mixed-gender: 47.7%; same-gender: 52.9%), and 44.1% (mixed-gender) and 37.4% (same-gender) were working professionals (see the Electronic Supplementary Material [ESM] A for a full breakdown of educational and occupational status). Note that we also collected data of same-gender couples containing men, but only 25 couples participated. Considering the small sample size, we only analyzed the couples consisting of women.

### Procedure

We advertised the study on campus, through social media, via leaflets, and on the authors’ department website as “a study of personality in romantic relationships,” with a link to our online questionnaire (hosted on www.soscisurvey.de). We asked participants to complete the questionnaire independently from their partner and to forward the link to their partner. There was no financial compensation, but psychology students could earn course credit by participating. Inclusion criteria for the study were speaking German, being ≥ 18 years of age, being in a couple, and being willing to forward the questionnaire to one’s partner. Data collection took place from November 2021 (after the German government lifted restrictions and the state of “epidemic situation of national scope” due to the COVID-19 pandemic) to July 2023. Our study was conducted in accordance with the World Medical Association's Declaration of Helsinki. The participants provided their informed consent to participate in this study. This type of study is exempt from ethics approval in Germany, as conducting psychological studies collecting questionnaire data are guided by the ethical guidelines of the German Psychological Association (https://www.dgps.de/fileadmin/user_upload/PDF/Berufsetische_Richtlinien/BER-Foederation-20230426-Web-1.pdf). We adhered to these guidelines.

### Instruments

#### Playfulness

The 28-item *OLIW Questionnaire*^1^ assesses four facets of playfulness in adults, namely, *Other-directed* (“I can express my feelings towards my romantic partner in a playful way”), *Lighthearted* (“Many people take their lives too seriously; when things don’t work you just have to improvise”), *Intellectual* (“If I want to develop a new idea further and think about it, I like to do this a playful manner”), and *Whimsical* (“I have the reputation of being somewhat unusual or flamboyant”). Each scale comprises seven items and participants give their responses on a 7-point Likert-type rating scale (1 = *strongly disagree*, 7 = *strongly agree*). There is robust evidence for the reliability (internal consistency, test–retest correlations ≥ 0.67 and ≥ 0.74 for 3- and 1-month intervals), factorial validity, including measurement invariance and agreement between self- and peer reports and between countries, associations with daily behavior ratings, and nomological validity^1,33,43–45^.

#### Attachment styles

We used the *Experiences in Close Relationships scale* (ECR^46^; German translation:^27^) to assess romantic attachment styles. The ECR questionnaire contains the scales *Anxiety* (i.e., worries about the relationship) and *Avoidance* (i.e., avoiding closeness and maintaining autonomy), with 18 items each. Sample items are “I am afraid that I will lose my partner’s love” (anxiety) and “I prefer not to be too close to romantic partners” (avoidance). Respondents rate their agreement on a 7-point Likert scale (1 = *completely disagree*, 7 = *completely agree*). The ECR is the standard instrument to assess attachment styles, and Neumann et al.^27^ provided findings on the German ECR’s reliability (e.g., αs ≥ 0.85) and validity, its robust two-factor structure, and its convergent and external validity (for a replication, see^28^).

#### Romantic jealousy

We used the *Multidimensional Jealousy Scale* (MJS^38^) to assess cognitive, emotional, and behavioral facets of romantic jealousy, with eight items each. *Cognitive* (e.g., “I think my partner is secretly developing a relationship with someone of the opposite sex”) and *Behavior* (e.g., “I question my partner about his or her whereabouts”) were rated on a scale from 1 (*never*) to 7 (*all the time*). *Emotional* scale items (e.g., “Your partner is flirting with someone of the opposite sex”) were rated from 1 (*very pleased*) to 7 (*very upset*). As the MJS was designed for opposite-sex relationships, we reformulated the items slightly to make them applicable for all couple types (e.g., “I think my partner is secretly developing a relationship with someone else”). The MJS is characterized by good psychometric properties, including good internal consistency (αs ≥ 0.85) and retest reliability (*r*_cog/emot/beh_ = 0.75/0.82/0.34 for 1–2-month intervals; recent findings show retest-correlations ≥ 0.71 for 5–9-month intervals^50^, its three-factor structure, and its convergent and discriminant validity^38,47,48^.

### Data analysis

**Fig. 1:**
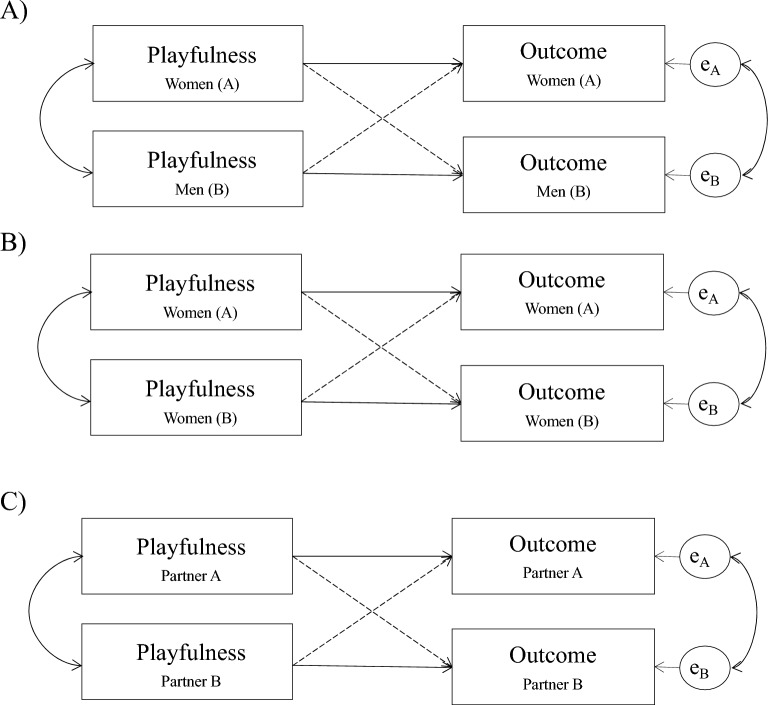
Actor-partner interdependence models (APIM) showing the associations between playfulness and the outcomes of romantic jealousy and romantic attachment. Model A = APIM for the sample of mixed-gender couples, with women being assigned to the role of Partner A and men being assigned to the role of Partner B. Model B = APIM for the sample of same-gender couples, with partners of the womens’ couples being randomly assigned to the role of Partner A and B. Model C = Fully constrained APIM assuming no differences between Models A and B, and thus, between partners and samples. Note that all data (except for the association between Other-directed playfulness behavioral jealousy) supported the assumption of Model C, with no distinction between samples and partners. Solid line: Actor effect. Dashed line: Partner effect.

We analyzed the data with the APIM^25^ to examine the associations between playfulness and romantic outcomes (attachment and jealousy). The APIM accounts for the interdependence between partners’ scores in predictor and outcome variables. As shown in Fig. 1, the APIM estimates the actor effects (associations between playfulness and jealousy/attachment within each partner) and partner effects (i.e., associations between partner A’s playfulness and B’s romantic outcome). Moreover, the APIM allowed us to examine whether actor- and partner-effects differed between partners by testing model constraints in which the actor-effects, as well as the partner effects, were constrained to equality between partners. A χ^2^ likelihood-ratio test was used to compare the model fit between the saturated (i.e., no equality constraints) and constrained models^25^. A non-significant test indicated that there was no robust difference between models and the parsimonious model was accepted. Note that in conventional APIMs, in analyzing data from mixed-gender couples the partners are differentiated by gender and constraints are set to examine whether gender played a role in the actor- and partner-effects. In same-gender couples, the partners were randomly assigned to be partner A and B, and as in mixed-gender couples, the model constraints were introduced to examine the statistical invariance between actor and partner effects between partners^49^. Additionally, we included constraints between our samples to examine whether the actor- and partner effects differed between same-gender and mixed-gender couples. Hence, a non-significant test would indicate that findings are invariant across partners of mixed-gender and same-gender couples. We computed APIM analyses in Mplus 8.8^50^. In line with the literature, we report unstandardized regression coefficients (*b*) and tested their statistical significance with Wald tests (i.e., computing a *z*-value by dividing the *b*-coefficient by its standard error). Hence, depending on their standard errors, two numerically identical *b* coefficients can differ in their statistical significance. In the ESM, we additionally report 95% confidence intervals (CIs) for all analyses. The CIs, standard errors, and *p*-values were computed based on 5000 bootstrapped random samples.

Our sample sizes met the recommendations for APIM analyses^25^. A power analysis with the APIMPoweR software^51^ showed that our sample allowed us to identify actor effects (β = 0.15) and partner effects (β = 0.10) with a power of 0.997 and 0.878, respectively.

## Results

### Preliminary analyses

The descriptive statistics, presented in ESM B, aligned with previous research findings in both samples^20–22^. Furthermore, the internal consistencies for all measures and samples were between 0.60 and 0.89, also comparable to earlier research^21,28,40^. As in prior research, we found the typical gender differences in mixed-gender couples, with men yielding higher scores in Lighthearted, Intellectual, and Whimsical playfulness (*d*s = 0.63, 0.26, and 0.40). Mean differences between partners in same-gender couples were negligible (*d*s ≤ 0.09). Our between-sample comparison showed no robust differences in playfulness, attachment, and behavioral and cognitive jealousy. In line with previous research comparing mixed- and same-gender couples in jealousy, the women in same-gender relationships were less emotionally jealous than the men (*d* = 0.95) and the women (*d* = 1.01) in mixed-gender couples.

Our examination of partner similarities (see ESM B) found that the mixed-gender couples showed comparable coefficients like in prior dyadic studies (e.g.,^21^), with *r*s = 0.29 (Other-directed), 0.17 (Intellectual), 0.29 (Whimsical), and Lighthearted playfulness showing no similarity (0.00). The same-gender couples showed comparable similarity in Lighthearted (*r*s = − 0.02) and Whimsical playfulness (0.24), but numerically lower similarity in Other-directed (0.13) and no similarity in Intellectual playfulness (− 0.02). For jealousy and attachment, we found the typical robust similarities (*r*s ≤ 0.44) and, in line with the literature, no similarity for attachment anxiety (0.03) in both samples.

### Actor-partner interdependence model analyses

Table 1 gives the regression coefficients of the APIM analyses that tested the associations between the four facets of playfulness and attachment styles and jealousy (see ESM C and D for exact *p*-values, CIs, and model tests). Our invariance analyses showed that the actor- and partner effects did not differ between the mixed-gender and same-gender couples (χ^2^ ≤ 12.3, *p* ≥ 0.06), except for Other-directed playfulness and behavioral jealousy, χ^2^ = 18.2, *p* = 0.006. Thus, actor- and partner effects between playfulness and attachment styles and jealousy were invariant between men and women in same-gender and mixed-gender couples (with one exception).

**Table 1:** Regression coefficients from multigroup actor-partner interdependence models testing associations between playfulness and romantic attachment styles and jealousy in mixed-gender and same-gender couples.

	Actor effects				Partner effects			
	Other-directed	Lighthearted	Intellectual	Whimsical	Other-directed	Lighthearted	Intellectual	Whimsical
Attachment styles								
Avoidance	− 0.14***	**− **0.01	**− **0.05	0.01	**− **0.03	0.04	**− **0.01	0.04
Anxiety	0.00	− 0.12***	− 0.10*	0.02	**− **0.01	0.04	0.00	0.06
Jealousy								
Cognitive	**− **0.02	0.04	**− **0.04	0.10**	0.01	0.06*	0.04	0.05
Emotional	− 0.11**	− 0.11**	− 0.21***	− 0.12**	**− **0.03	0.00	**− **0.06	− 0.08*
Behavioral	0.04^a^/0.10^b^/0.01^c^	0.04	0.01	0.06*	**− **0.09^a^/0.08^b^/0.00^c^	0.02	**− **0.02	0.03

*N* = 471 couples (*n* = 332 mixed-gender; 139 same-gender). *p < .05. **p < .01. ***p < .001. Two-tailed. Statistically significant coefficients bold. ^a/b/c^Coefficients for men in mixed-gender couples/women in mixed-gender couples/women in same-gender couples.

#### Attachment styles

As expected, Other-directed playfulness was negatively associated with avoidant attachment in actors (*b* = − 0.14, 95% CI [− 0.20, − 0.08], *p* < 0.001), but contrary to expectations, we found no relationship with attachment anxiety, *b* = 0.00, 95% CI [− 0.07, 0.08], *p* = 0.909. Hence, our expectations for Other-directed playfulness were only partially met. However, Lighthearted playfulness was characterized by low attachment anxiety, *b* = − 0.12, 95% CI [− 0.19, − 0.06], *p* < 0.001. Furthermore, Intellectual playfulness related to lower attachment anxiety, *b* = − 0.10, 95% CI [− 0.19, − 0.02], *p* = 0.015, whereas Whimsical playfulness was unrelated to attachment styles. Finally, facets of playfulness were unrelated to partners’ attachment (*b*s ≤ |0.06|, *p* ≥ 0.091).

#### Jealousy

We found differential actor effects for playfulness and jealousy: all types of playfulness related to less intense and less frequent emotional reactions to threats of relationships, with *b*s between − 0.11 (Whimsical) and − 0.21 (Intellectual; 95% CIs_UL_ ≤ − 0.04, *p*s ≤ 0.002). Conversely, Whimsical playfulness related positively to cognitive (*b* = 0.10, 95% CI [0.03, 0.16], *p* = 0.003) and behavioral jealousy (*b* = 0.06, 95% CI [0.01, 0.11], *p* = 0.015). As expected, our analyses of partner effects showed that Lighthearted playfulness related to greater cognitive jealousy in their partners, *b* = 0.06, 95% CI [0.01, 0.12], *p* = 0.017. Furthermore, Whimsical playfulness related to the partners reporting less emotional jealousy, *b* = − 0.08, 95% CI [− 0.15, − 0.01], *p* = 0.029.

As noted, actor- and partner effects between Other-directed playfulness and behavioral jealousy differed regarding gender and the couples’ gender composition, χ^2^ = 18.2, *p* = 0.006. However, the inspection of the APIM parameters showed that neither men nor women from same-gender or mixed-gender couples yielded robust actor- and partner effects (all *b*s ≤ 0.10, 95% CIs including zeros). Hence, although there was a statistical difference between parameters, none of the associations were robust upon further analysis.

## Discussion

Our study aimed to narrow the gap in the literature on playfulness in romantic relationships in two ways. First, we advanced the understanding of how playfulness relates to two of the most studied variables in relationship research—romantic jealousy and attachment. These two variables are key to understanding individual differences in how people experience their relationships^30,38^. Second, we collected data from both same-gender and mixed-gender couples. This allowed us to address whether expressions of playfulness and their relationships to indicators of romantic life are invariant between couples of different gender compositions.

### Playfulness in mixed-gender and same-gender couples

To our knowledge, this was the first study examining dyadic data of same-gender and mixed-gender couples. Previous studies on playfulness in couples had limited generalizability, as only mixed-gender couples were studied, and it remained unclear whether playfulness is expressed differently in same-gender couples^3,20–22^. Our findings revealed no robust mean-level differences in playfulness based on couple’s gender composition. However, variability *within* and between partners did differ. In mixed-gender couples, men tended to score slightly higher on Lighthearted, Intellectual, and Whimsical playfulness compared to their female partners. We did not observe this pattern of differences in the same-gender couples. Overall, we found no robust mean differences between our samples. Hence, expressions of playfulness in couples were unrelated to gender composition, but variation *within* couples and between partners differed in the expected way. The mixed-gender couples showed the typical mean differences, with men showing, on average, slightly higher scores in Lighthearted, Intellectual, and Whimsical playfulness, whereas this pattern did not exist between the women in same-gender couples. Furthermore, our analysis of partner similarities showed that the similarity coefficients were comparable between samples per Lighthearted, Whimsical, and to some degree Other-directed playfulness. Contrary to mixed-gender couples from the present and prior studies, we found negligible similarity for Intellectual playfulness in our same-gender couples. Thus, while average expressions were comparable between mixed-gender and same-gender samples, the partner similarity regarding the facet of Intellectual playfulness could indicate differences. It could be argued that gender differences might play a role in the similarity correlations, because when women are, on average, like each other, there is less room for variability that allows for meaningful matching between partners at different *levels* of playfulness. It is important to note that these findings require replication, particularly by including same-gender couples comprising men. In test of our hypothesis, we would expect to see a similar pattern in this subgroup: minimal average score differences between partners and relatively low similarity correlations.

From a theoretical perspective, our findings raise questions about the desirability and role of playfulness during mating. The Signal Theory of Playfulness^16^ suggests that playfulness signals underlying qualities of non-aggressiveness and fecundity in the opposite sex. It remains unclear what playfulness might also indicate for (or signal about) same-gender constellations, and whether the desirability of playfulness differs between same-gender versus mixed-gender couples. Future research should address these questions by testing same-gender couples consisting of men and women. Interpretations of our data regarding similarities and mean differences should be preliminary because we only collected self-reports of playfulness. We argue that additional partner-reports and ratings of desirability are needed to create a more comprehensive picture of the role of gender differences and similarities in playfulness in couples, which would allow for conclusions about the descriptive and normative similarities and differences of playfulness in same-gender and mixed-gender couples. Such findings could help extend the Signal Theory of Playfulness to same-gender romantic relationships.

### Localization of playfulness in systems of romantic attachment and jealousy

Although playfulness has received strong interest regarding its role in adult relationships, our study is the first to present initial findings on how playfulness relates to jealousy and attachment. When comparing the average expressions across samples and gender, we replicated prior research showing that women from same-gender couples report, on average, lower jealousy expressions than men and women from mixed-gender couples^52,53^. Also, in line with prior research (e.g.,^54^), attachment expressions did not differ by gender or gender composition. Hence, these prior findings replicated well in German-speaking couples.

Using data from same-gender and mixed-gender couples allowed us to address the question whether actor- and partner effects depend on couples’ gender composition. Overall, our findings were invariant regarding gender (within couples) and gender composition (between couples), contributing to the generalizability of findings on playfulness in relationships. We hope that our study stimulates future research that extends the knowledge on playfulness in couples by replicating prior research on variables such as relationship satisfaction in same-gender couples, and by further examining the descriptive differences in, for example, partner similarities. As discussed, such research will ideally include partner ratings.

#### Romantic attachment

Attachment styles describe expectations, behaviors, and attitudes toward relationships and predict major indicators of romantic life, such as relationship satisfaction and the likelihood of entering a relationship^29,32^. As expected, Other-directed playfulness correlated negatively with avoidant attachment. This means people who enjoy using playfulness in their relationships, like playful teasing, tend to be less inclined to emotionally distance themselves from their partners. This allows for greater intimacy, which aligns with Fraley and Roisman's^30^ findings and fits with literature that suggested playfulness fulfills a social function, as it enhances bonding and trust in primates and humans^3,55^. However, contrary to expectations, Other-directed playfulness was independent from anxious attachment. Hence, although those high in Other-directed playfulness have been characterized by expressing low avoidant attachment, some partners show inclinations to worry about whether their affection to their partner is reciprocated and are sensitive to signs of rejection^30^. Our initial expectation that Other-directed playfulness would be associated with a secure attachment style (low avoidance and anxiety) was only partially supported. While playfulness generally contributes to positive relationships by fostering bonding, intimacy, and trust, we found no direct link between Other-directed playfulness and relationship anxiety in actors.

As expected, Lighthearted playfulness related to lower inclinations in attachment anxiety. Thus, the low worrying and a preference for improvisation, rather than planning that characterizes Lighthearted playfulness, translated into showing less relationship-specific worries. This fits with prior findings on Lighthearted playfulness relating negatively to indicators of detachment and the literature characterizing Lighthearted as a predictor of entering relationships^36,43^.

Our exploratory analyses showed that Intellectual playfulness was also negatively related to attachment anxiety. Perhaps those higher in Intellectual playfulness can use their playfulness to deal with situations that prompt relationship worries in proactive ways that allow them to regulate anxiety. This finding might shed light on why Intellectual playfulness frequently emerges as a strong predictor of relationship satisfaction^21,22^. Attachment styles are robust mediator variables that can (partially) explain the link between personality traits and relationship satisfaction. Similarly, attachment might help explain the association between Intellectual playfulness and satisfaction via indirect effects.

As in other studies that examined couples’ attachment dimensions, we did not find partner effects. However, longitudinal research might reveal partner effects because playfulness can build trust over time^3^ and attachment styles are malleable through learning experiences in relationships^56^. Hence, we expect that playfulness might relate to one’s partner’s attachment style when studied over time or could predict change in one’s partner’s attachment style. For example, we would expect that those with less positive working models of relationships (insecure attachment) could benefit from having a partner high in Other-directed playfulness, showing their affection in creative and playful ways (e.g., by solving conflict through re-playing shared experiences, teasing each other in a loving way, and giving nicknames). At the same time, prior research has shown that partners of Lighthearted high-scorers report mistrust^21,22^, and it is possible that this contributes to negative relationship experiences of partners, which could relate to an increase in relationship worries.

From a practical perspective, our findings might be used in couples’ therapy when addressing issues around relationship and sexual satisfaction^14,15^. They might also be of use in individual therapy. Established playfulness training programs with short daily exercises are available^36,57^, and future research could examine their effects on attachment in individuals and couples. Considering that insecure attachment is a robust predictor of long-term singlehood^29,32,58^, testing the bidirectional effects of playfulness and attachment might be especially beneficial for long-term singles with pronounced expressions of insecure attachment. Taking the effects of playfulness programs on depression and well-being and the present findings into account, we would expect that playfulness programs could help change attachment styles and alleviate the negative consequences of insecure attachment.

#### Romantic jealousy

Our analyses of playfulness and jealousy highlight the importance of examining fine-grained facets, as we found differential associations of minor effect sizes. We found that all types of playfulness related negatively to emotional jealousy in actors. Whimsical playfulness also yielded higher expressions in cognitive and behavioral types of jealousy. Overall, our findings expand the knowledge on actors’ experiences of jealousy in comparison to prior research^21,22^, which examined mistrust toward the partner as part of a satisfaction measure and found no associations with the OLIW facets, indicating that playfulness might operate independently from jealousy. Our findings, however, show that this was true for cognitive and behavioral types of jealousy, whereas those high in playfulness show less inclination to being upset in reaction to situations that could be interpreted as a threat to one’s relationship^38^. While we cannot draw conclusions about the mechanism behind this finding, it could be argued, in line with Fredrickson’s^59^ Broaden-and-Build Theory, that playfulness contributes to the experience of positive emotions through several pathways. Playfulness might also buffer negative emotional reactions to some degree. Additionally, some research with children found that playfulness supports learning the notion that close others can be trusted. While the link between playfulness and emotion regulation strategies has not yet been tested, it might help provide one potential explanation for why playfulness has related negatively to emotional jealousy. The Signal Theory of Playfulness might provide an explanation for these findings (i.e., playfulness as a signal of low aggression), while jealousy has been linked to aggressive behaviors in close relationships^60,61^. This is in line with the finding that Whimsical playfulness relates to both cognitive and behavioral jealousy. Notably, Whimsical playfulness has been the only facet linked to self-reported aggression (including daily diary entries aggregated over 14 days) with similar effect sizes as those observed for cognitive and behavioral jealousy. However, more research is warranted to clarify this and the underlying mechanisms.

As expected, partners of those high in Lighthearted playfulness reported greater cognitive jealousy and worries about their partner’s infidelity. This fits well with the prior research that showed that partners of Lighthearted high scorers reported greater mistrust^21,22^. The literature noted that partners might perceive the preferences for spontaneity over planning in everyday life and the liking of improvisation that characterizes Lighthearted playfulness as a potential sign of non-commitment in relationships, which contributes to mistrust and cognitive jealousy of their partners. Using the multidimensional approach to jealousy allowed us to clarify which type of jealousy was evoked in partners: We found that these were focused on worries and cognitive reactions versus emotional and behavioral inclinations of jealousy. Against expectations, the partners of those high in Whimsical playfulness reported less emotional jealousy. Interestingly, the actor effects of playfulness on emotional jealousy translated to partners regarding the negative direction, but (except for Whimsical playfulness) they did not reach statistical significance. This might indicate that playfulness and jealousy are one of the few cases in which actor effects translate to partner effects, and the latter are typically smaller in size^62^. However, given the small effect sizes, its practical significance should not be overstated.

Overall, our findings do not suggest that greater playfulness necessarily relates to dysregulation in reframing processes as reported in clinical samples of patients with anxiety disorder^42^. Our study focused on non-clinical populations. Examining jealousy and attachment in clinical samples might offer insights into more extreme expressions of these variables. However, such samples might be more heavily composed of individuals who are not currently in partnerships^29,32^. As discussed regarding attachment styles, longitudinal analyses and studies of programs/interventions to increase playfulness in individuals and couples could further enhance our knowledge about the pathways of playfulness to experiences in romantic relationships.

### Limitations and future directions

Our findings must be interpreted in the light of study limitations. Since our study employed a cross-sectional design, we cannot establish causal relationships between playfulness, jealousy, and attachment styles. To better understand the directionality of these findings and potential underlying mechanisms, longitudinal research is necessary. This would allow us to explore how playfulness in a romantic relationship might influence actors’ and partners’ experiences of jealousy and attachment over time. Additionally, longitudinal data would allow the examination of the dynamics between jealousy and attachment, as complex interactions between the activation of internal working models of relationships and jealousy may exist. Furthermore, we did not assess sexual orientation and, thus, cannot clarify whether the participants in the subsamples were, for example, heterosexual or homosexual. Hence, the exclusiveness of gender and gender composition was only assumed but not given because participants might be bisexual. Moreover, an issue of all studies testing attachment in couples is that generalizability is limited because those who enter relationships show, on average, a more secure attachment style than long-term singles^29^. Also, our sample of same-gender couples consisted exclusively of women, and we cannot generalize the findings to same-gender couples consisting of men or other non-binary genders. Future research is needed to further extend our findings’ generalizability. Additionally, while our main findings did not differ between samples, it is important to note that the relationship lengths varied across samples. Participants in both samples exhibited relationship durations suggesting stability^63^. Future research should examine the role of relationship duration on the associations between playfulness, jealousy, and attachment. Following best practices, we recommend analyzing relationship duration with longitudinal data, because research indicates that cross-sectional analyses of relationship duration as a between-couple variable can lead to biased results (opposed to testing within-dyad variability over time; see^64^, for an in-depth discussion). Finally, we relied on self-reports only and the inclusion of additional data sources, such as partner reports or diary data, is desirable to both reduce shared method variance and overcome biases^65^.

## Conclusion

Our findings expand the knowledge about playfulness in romantic life in several ways by extending the nomological net in romantic life and by addressing whether findings are invariant between same-gender and mixed-gender couples. We hope our findings inspire further research into the multifaceted role of playfulness in romantic relationships. The comparative approach across same-gender and opposite-gender couples offered a valuable avenue to not only expand our understanding of playfulness within the existing nomological net of variables, but also to explore its nuances in diverse relationship types.

### Supplementary Information

Supplementary Tables.

## Data Availability

All data and syntaxes underlying this research are openly available in the Open Science Framework under osf.io/h6xrm/?view_only=7bf15dc2e5504333bfe7ec8047534c27.
