# Supplementary material for: Extending the study of playfulness in romantic life: Analyzing associations with attachment and jealousy in same-gender and opposite-gender couples
Source: Sci Rep. 2024 Aug 30;14:20190. doi: 10.1038/s41598-024-70979-2 (PMC11364825; doi:10.1038/s41598-024-70979-2)
Supplement: Supplementary file 1 — Supplementary Tables. [file 41598_2024_70979_MOESM1_ESM.docx]

**Electronic Supplementary Materials**

**Table A**

*Sample Descriptions*

|  | Mixed-Gender Couples | Same-Gender Couples |
| --- | --- | --- |
| Age | *M* = 29.30 years (*SD* = 11.80) | *M* = 27.26 years (*SD* = 7.36) |
| Occupation  University students  Working professionals  In vocational training  Unemployed  Retired  Other | 47.7%  44.1%  3.0%  1.5%  1.8%  1.8% | 52.9%  37.4%  5.4%  2.2%  0.4%  1.8% |
| Education  9 years of school  10 years of school  12–13 years of school  Vocational training  University degree  Other | 0.9%  7.0%  40.1%  10.8%  40.5%  0.8% | 0.7%  2.2%  39.9%  9.4%  47.8%  0.0% |
| Relationship duration | *M* = 6.77 years (*SD* = 9.72),  *Mdn* = 3.33 years | *M* = 2.85 years (*SD* = 3.73),  *Mdn* = 1.75 years |
| Living together  No  At times (e.g., weekend)  Yes | 27.7%  15.2%  57.1% | 42.1%  10.8%  42.1% |
| Marriage | 20.5% were married | 10.8% were married |

*Note. N* = 332 (mixed-gender) and 139 (same-gender couples).

**Table B**

*Descriptive Statistics, Partner Similarities (*r*), and Mean Differences between Partners (Cohen’s d Effect Size)*

|  | Mixed-gender couples (*N* = 332 couples) | | | | | |  | Same-gender couples (*N* = 139 couples) | | | | |
| --- | --- | --- | --- | --- | --- | --- | --- | --- | --- | --- | --- | --- |
|  | α | *M*_M/W_ | *SD*_M/W_ |  | *r* | *d* |  | α | *M* | *SD* | *d* | *r* |
| Playfulness |  |  |  |  |  |  |  |  |  |  |  |  |
| Other-directed | .72 | 5.25 / 5.19 | 0.89 / 0.90 |  | .29 | 0.07 |  | .66 | 5.18/5.23 (5.21) | 0.85 | 0.06 | .13 |
| Lighthearted | .79 | 4.28 / 3.65 | 1.01 / 0.98 |  | .00 | 0.63 |  | .81 | 3.83/4.03 (3.93) | 1.09 | 0.19 | -.02 |
| Intellectual | .59 | 4.15 / 3.95 | 0.83 / 0.77 |  | .17 | 0.26 |  | .60 | 4.07/4.12 (4.09) | 0.81 / 0.79 | 0.07 | -.02 |
| Whimsical | .74 | 4.15 / 3.78 | 0.91 / 0.95 |  | .21 | 0.40 |  | .73 | 4.17/4.21 (4.19) | 0.96 / 0.89 | 0.04 | .24 |
| Attachment |  |  |  |  |  |  |  |  |  |  |  |  |
| Avoidance | .84 | 2.41 / 2.24 | 0.71 / 0.75 |  | .13 | 0.23 |  | .84 | 2.22 | 0.70 |  | .35 |
| Anxiety | .86 | 3.18 / 3.50 | 0.90 / 1.01 |  | .03 | -0.33 |  | .86 | 3.32 | 0.94 |  | .03 |
| Jealousy |  |  |  |  |  |  |  |  |  |  |  |  |
| Cognitive | .85 | 2.24 / 2.05 | 0.91 / 0.96 |  | .30 | 0.20 |  | .82 | 2.02 | 0.86 |  | .38 |
| Emotional | .87 | 4.34 / 4.53 | 0.96 / 1.01 |  | .28 | -0.19 |  | .89 | 3.35 | 1.14 |  | .44 |
| Behavioral | .75 | 1.93 / 2.15 | 0.69 / 0.81 |  | .32 | -0.29 |  | .72 | 1.80 | 0.64 |  | .43 |

**Table C**

*Multigroup APIM Analyses with Playfulness Predicting Attachment in Mixed-Gender and Same-Gender Couples*

|  | Actor effects | | | |  | Partner effects | | | |  | Model test | |
| --- | --- | --- | --- | --- | --- | --- | --- | --- | --- | --- | --- | --- |
|  | *b* | 95% CI | *p* | \|Δ_1/2/3_\| |  | *b* | 95% CI | *p* | \|Δ_1/2/3_\| |  | χ^2^ | *p* (χ^2^) |
| Avoidance |  |  |  |  |  |  |  |  |  |  |  |  |
| Other-directed | **-0.14** | [-0.20, -0.08] | < .001 | 0.20 / 0.19 / 0.20 |  | -0.03 | [-0.09, 0.02] | .238 | 0.04 / 0.05 / 0.05 |  | 4.10 | .663 |
| Lighthearted | -0.01 | [-0.06, 0.04] | .565 | 0.02 / 0.02 / 0.02 |  | 0.04 | [-0.01, 0.08] | .129 | 0.05 / 0.05 / 0.05 |  | 6.16 | .406 |
| Intellectual | -0.05 | [-0.11, 0.01] | .123 | 0.07 / 0.07/ 0.07 |  | -0.01 | [-0.07, 0.05] | .726 | 0.01 / 0.01 / 0.01 |  | 8.87 | .181 |
| Whimsical | 0.01 | [-0.04, 0.07] | .598 | 0.02 / 0.02 / 0.02 |  | 0.04 | [-0.01, 0.09] | .136 | 0.05 / 0.05 / 0.06 |  | 3.27 | .775 |
| Anxiety |  |  |  |  |  |  |  |  |  |  |  |  |
| Other-directed | 0.00 | [-0.08, 0.07] | .909 | 0.00 / 0.00 / 0.00 |  | -0.01 | [-0.08, 0.06] | .768 | 0.01 / 0.01 / 0.01 |  | 10.23 | .115 |
| Lighthearted | **-0.12** | [-0.19, -0.06] | < .001 | 0.14 / 0.12 / 0.13 |  | 0.04 | [-0.02, 0.09] | .244 | 0.03 / 0.04 / 0.04 |  | 3.27 | .774 |
| Intellectual | **-0.10** | [-0.19, -0.02] | .015 | 0.12 / 0.10 / 0.11 |  | 0.00 | [-0.08, 0.08] | .921 | 0.00 / 0.00 / 0.00 |  | 3.68 | .720 |
| Whimsical | 0.02 | [-0.05, 0.09] | .586 | 0.02 / 0.02 / 0.02 |  | 0.06 | [-0.01, 0.13] | .091 | 0.06 / 0.07 / 0.06 |  | 12.27 | .056 |

*Note.* df for χ^2^ model test = 6. *N* = 332 mixed-gender couples and *N* = 139 women same-gender couples. Δ = *b*/*SD*, based on the subgroups’ *SD.* Standard errors, *p*-values, and 95% confidence intervals (CI) based on 5,000 bootstrap samples.

**Table D**

*Multigroup APIM Analyses with Playfulness Predicting Jealousy in Mixed-Gender and Same-Gender Couples*

|  | Actor effects | | | |  | Partner effects | | | |  | Model test | |
| --- | --- | --- | --- | --- | --- | --- | --- | --- | --- | --- | --- | --- |
|  | *b* | 95% CI | *p* | \|Δ_1/2/3_\| |  | *b* | 95% CI | *p* | \|Δ_1/2/3_\| |  | χ^2^ | *p* (χ^2^) |
| Cognitive Jealousy |  |  |  |  |  |  |  |  |  |  |  |  |
| Other-directed | -0.02 | [-0.09, 0.06] | .667 | 0.02 / 0.02 / 0.02 |  | 0.01 | [-0.06, 0.08] | .814 | 0.01 / 0.01 / 0.01 |  | 2.38 | .882 |
| Lighthearted | 0.04 | [-0.01, 0.10] | .129 | 0.05 / 0.04 / 0.05 |  | **0.06** | [0.01, 0.12] | .017 | 0.07 / 0.07 / 0.07 |  | 3.46 | .750 |
| Intellectual | -0.04 | [-0.11, 0.04] | .351 | 0.04 / 0.04 / 0.04 |  | 0.04 | [-0.03, 0.11] | .226 | 0.04 / 0.05 / 0.05 |  | 4.06 | .670 |
| Whimsical | **0.10** | [0.03, 0.16] | .003 | 0.11 / 0.10 / 0.11 |  | 0.05 | [-0.01, 0.12] | .094 | 0.05 / 0.06 / 0.06 |  | 7.22 | .301 |
| Emotional Jealousy |  |  |  |  |  |  |  |  |  |  |  |  |
| Other-directed | **-0.11** | [-0.18, -0.04] | .002 | 0.12 / 0.11 / 0.10 |  | -0.03 | [-0.10, 0.04] | .371 | 0.03 / 0.03 / 0.03 |  | 4.98 | .547 |
| Lighthearted | **-0.11** | [-0.17, -0.04] | .001 | 0.11 / 0.11 / 0.09 |  | 0.00 | [-0.07, 0.06] | .903 | 0.00 / 0.00 / 0.00 |  | 5.18 | .521 |
| Intellectual | **-0.21** | [-0.30, -0.13] | < .001 | 0.22 / 0.20 / 0.18 |  | -0.06 | [-0.15, 0.02] | .151 | 0.06 / 0.06 / 0.05 |  | 3.29 | .772 |
| Whimsical | **-0.12** | [-0.19, -0.05] | .001 | 0.12 / 0.12 / 0.10 |  | **-0.08** | **[-0.15, -0.01]** | **.029** | **0.08 / 0.08 / 0.07** |  | 8.62 | .196 |
| Behavioral Jealousy |  |  |  |  |  |  |  |  |  |  |  |  |
| Other-directed  MGC: Men  MGC: Women  SGC: Women | 0.04  0.10  0.01 | [-0.06, 0.14]  [<0.00, 0.19]  [-0.11, 0.11] | .445  .055  .862 | 0.06  0.12  0.02 |  | -0.09  0.08  0.00 | [-0.19, 0.03]  [0.00, 0.15]  [-0.08, 0.09] | .113  .039  .999 | 0.11  0.11  0.01 |  | **18.17** | .006 |
| Lighthearted | 0.04 | [-0.01, 0.09] | .106 | 0.06 / 0.05 / 0.06 |  | 0.02 | [-0.02, 0.07] | .271 | 0.03 / 0.03 / 0.04 |  | 5.10 | .532 |
| Intellectual | 0.01 | [-0.04, 0.06] | .767 | 0.01 / 0.01 / 0.01 |  | -0.02 | [-0.07, 0.04] | .523 | 0.02 / 0.02 / 0.03 |  | 5.27 | .510 |
| Whimsical | **0.06** | [0.01, 0.11] | .015 | 0.09 / 0.08 / 0.10 |  | 0.03 | [-0.02, 0.08] | .221 | 0.04 / 0.05 / 0.05 |  | 6.17 | .405 |

*Note.* df for χ^2^ model test = 6. *N* = 332 mixed-gender couples (MGC) and *N* = 139 female same-gender couples (SGC). Δ = *b*/*SD*, based on the subgroups’ *SD.* Standard errors, *p*-values, and 95% confidence intervals (CI) based on 5,000 bootstrap samples. 1/2/3 = Men in MGC / Women in MGC / Women in SGC.
